# Identifying dynamic reproducible brain states using a predictive modelling approach

**DOI:** 10.1162/imag_a_00540

**Published:** 2025-04-17

**Authors:** David O’Connor, Corey Horien, Francesca Mandino, Robert Todd Constable

**Affiliations:** Department of Biomedical Engineering, Yale University, New Haven, CT, United States; Yale MRRC Neuroscience, Yale School of Medicine, New Haven, CT, United States; Interdepartmental Neuroscience Program, Yale School of Medicine, New Haven, CT, United States; Department of Radiology and Biomedical Imaging, Yale School of Medicine, New Haven, CT, United States

**Keywords:** fMRI, brain behavior modeling, dynamic functional connectivity, generalizability

## Abstract

Conceptually, brain states reflect some combination of the internal mental processes of a person, and the influence of their external environment. Importantly, for neuroimaging, brain states may impact brain-based modeling of a person’s traits, which should be independent of moment-to-moment changes in behavior. Investigation of brain states, and modeling of traits or behaviors are both often done using fMRI-based functional connectivity. Brain states can fluctuate in time periods shorter than a typical fMRI scan, and an array of methods called dynamic functional connectivity analyses has been developed to measure them. It has previously been shown that brain state can be manipulated through the use of continuous performance tasks that put the brain in a particular configuration while the task is performed. Here, we focus on moment-to-moment changes in brain state and test the hypothesis that there are particular brain-states that maximize brain-trait modeling performance. We use a regression-based framework, Connectome-based Predictive Modelling, allied to a resample aggregating approach, to identify behavior and trait-related brain states, as represented by dynamic functional connectivity maps. We find that there is not a particular brain state that is optimal for trait-based prediction, and combining data from distinct brain states across the scan is better. We also find that this is not the case for in-scanner behavioral prediction where more isolated and temporally specific parts of the scan session are better for building predictive models of behavior. The resample aggregated dynamic functional connectivity models of behavior replicated in sample using unseen left-out data. The modeling framework also showed success in estimating variance in behavior in a separate dataset. The method detailed here may prove useful for both the study of behaviorally related brain states, and for short-time predictive modeling.

## 1 Introduction

Investigation of brain function using fMRI and functional connectivity (FC) has yielded increased insight into whole-brain functional topology and the relationship between brain function and behavior (Fox et al., 2005;Thomas Yeo et al., 2011). FC has been shown to be individually unique (Finn et al., 2015) and has been used to investigate and derive reproducible brain-behavior relationships (Rosenberg et al., 2018;Shen et al., 2017). The majority of FC-based studies aggregate information across an entire four-dimensional scan into a one- or two-dimensional “static” measure of FC, for example a graph theory metric (Sporns, 2018), or an adjacency matrix (Van Den Heuvel & Pol, 2011). While these metrics have provided insight into brain-behavior relationships, they are, by design, insensitive to events which occur on shorter time scales. This can obscure functional patterns present in the moment-to-moment data, which are cognitively relevant. It has been shown that FC topology varies on timescales much shorter than a typical scan length (Handwerker et al., 2012;Jones et al., 2012), and the characteristics of such variations may reflect inter-subject trait differences (Cabral et al., 2017;Vidaurre et al., 2017). Studies incorporating temporal variation in FC into their analyses are on the rise (Lurie et al., 2020). Such investigations use what has been described as time varying or dynamic functional connectivity (dFC).

It has been shown that the task a participant is engaged in during data acquisition can be determined from time windows as short as tens of seconds (Gonzalez-Castillo et al., 2015). Variations at this time-scale have been tied to changes in vigilance and cognitive engagement (G. J. Thompson et al., 2013); they can be used to predict human error (Eichele et al., 2008), to assess cognitive decline in an elderly population (Cabral et al., 2017), and to investigate short-time functional reorganization in healthy and diseased brains, for example, between typical and schizophrenic participants (Sakoğlu et al., 2010). Methods for estimating dFC have emerged in order to encapsulate as much data from one individual’s scan as possible (Calhoun et al., 2014;Chang & Glover, 2010;Hutchison et al., 2013;Lurie et al., 2020). With these dFC methods the goal is often to find brain “states”, which are temporal snapshots of connectivity thought to be relevant to a behavior, or a trait. One of the more common approaches involves using sliding window estimates of FC, which allow calculation of correlations between time series at shorter timeframes or “windows” (Sakoğlu et al., 2010). Other methods for deriving short-time FC include phase synchrony connectivity (Cabral et al., 2017;Glerean et al., 2012), tapered sliding windows, spatial distances, and temporal ICA; a partial list is detailed here (W. H. Thompson et al., 2018). Phase synchrony connectivity is of particular interest, as it allows derivation of “instantaneous” connectivity, which can be estimated close to the temporal resolution of the fMRI acquisition.

Once calculated, these windowed estimates of FC from different time points can be clustered into groups reflecting different functional connectivity configurations, or brain states. The prototypical approach byAllen et al. (2014)used*k*-means based clustering to accomplish this. Following clustering, dwell time within states and state-switching frequency can be calculated. These metrics have been considered as indicators of trait-based differences (Calhoun et al., 2014). Other brain state detection methodologies include Hidden Markov Modelling (Shappell et al., 2019;Vidaurre et al., 2017), change point estimates (Cribben et al., 2012;Xu & Lindquist, 2015), and dictionary learning based “windowless” connectivity (Yaesoubi et al., 2018). There are also methods for defining temporally overlapping brain states (W. H. Thompson & Fransson, 2015). The common hypothesis in these approaches is that there is a reproducible set of common spatiotemporal patterns that the brain transitions through during a given scan, which can provide insight into the cognitive processes ongoing during data acquisition.

While dFC allows the identification of temporally discrete FC, these metrics are based on fewer data points, and thus have lower SNR, poorer reliability, and are more susceptible to typical noise processes (Hutchison et al., 2013). While changes in resting state fMRI (rs-fMRI) based dFC may be the result of unconstrained cognition fluctuations (Doucet et al., 2011), it is also possible that sampling error, motion, and arousal can affect these changes (Laumann et al., 2017). Validation of dFC patterns is difficult, as we often lack a ground truth for what spurs changes in dFC. One approach to statistical validation of dFC changes is null modeling. Past dFC studies have constructed null spaces, simulating random data from real covariance matrices and power spectra (Laumann et al., 2017;Miller et al., 2018). Using these methods, it is possible to distinguish cognitively relevant dFC from noise patterns. Construction of null spaces is particularly relevant for studies involving resting state fMRI, which constitute the majority of dFC studies performed (Lurie et al., 2020). Another way to mitigate a lack of ground truth is to generate more constrained feature sets via low-dimensional representations of rs-fMRI dFC, which are inherently more stable (Kurtin et al., 2023;Sastry et al., 2023;Xie et al., 2019). However, this is often achieved by aggregating temporally precise dFC estimates via clustering or dimensionality reduction. This naturally results in a loss of temporal specificity but can result in increased stability/SNR (Hancock et al., 2022;Preti et al., 2017). An alternative to rs-fMRI is task-based fMRI (for a detailed review on the task/rest dichotomy, in the context of dFC, seeGonzalez-Castillo & Bandettini (2018)). Studies assessing noise dominance on dFC focus mostly on resting state fMRI (Laumann et al., 2017). Integrating task and rest dFC can improve confidence in the relationships estimated (Calhoun et al., 2014). More reproducible, and synchronous, changes in FC can be driven by giving participants a common task. Ultimately, null modeling, more constrained feature sets, and task-based dFC have shown the potential to validate dFC approaches.

Model-based approaches are frequently used to capture the relationship between static FC and behavior/traits. These methods allow for statistical validation via model building and testing. Even with some statistical validation, such as null modeling, it is important to test the generalization of any statistical relationship derived. One modeling method which has shown great promise in FC-based modeling is connectome-based predictive modelling (CPM) (Shen et al., 2017). CPM has been used to successfully predict attention (Rosenberg et al., 2018), abstinence from drug use (Yip et al., 2019), obesity (Farruggia et al., 2020), and ADHD/autism-related traits (Lake et al., 2019). CPM studies typically involve the application of models to unseen data, to ensure a level of reproducibility in the estimated relationship. A similarly rigorous approach can be used to assess the reproducibility of dFC-based models. Predictive modeling-based approaches have been used with dFC, though mostly classification-based, for example task classification (Gonzalez-Castillo et al., 2015), and disease classification (schizophrenia and bipolar disorder) (Rashid et al., 2016). Regression-based approaches to dFC brain-behavior modeling are few.Fong et al. (2019)used dFC estimates to model attention performance, though still aggregating data across whole scans into the model. Nonetheless, the success of both these model-based studies suggests that regression-based approaches, such as CPM, may have the potential to both identify brain states from short-time dFC and link them to specific behaviors or traits.

In this study, we use a combination of a regression model-based approach, CPM, and phase synchrony based dFC to identify specific temporal periods, or brain states, that are related to a working memory-based trait and short time-varying in-scanner behavior. Specifically, we seek to assess whether more behaviorally salient dFC estimates could be extracted by using time-varying behavior as a target in a regression-based predictive framework. We contrast the predictive power of rest and task-based fMRI, for modeling both trait and behavior, and show that task-based prediction of behavior may be more optimal for identifying temporally unique trait relevant brain states. We assess the models derived using cross-validation, within sample testing (in the Human Connectome Project (HCP) dataset) and out of sample testing (in the Adolescent Brain Cognitive Development (ABCD) dataset), showing good generalizability of these brain state models within the sample. Our study offers a framework for identifying and validating behavior-related brain states using predictive modeling.

## 2 Methods

### 2.1 Datasets

Data from the HCP dataset, specifically the S900 dataset (Van Essen et al., 2013) and the ABCD dataset (Casey et al., 2018), from the Fast-track Imaging Data Release (year one arm), were used. From these datasets, N = 827 participants from the HCP, ages 21–35, N = 1,668 ABCD participants from the ABCD, ages 9–11, were included. HCP data were collected at a single site while ABCD data were collected across 21 sites. Inclusion criteria for both data sets were based on the availability of preprocessed T_1_-weighted images, N-Back task fMRI scans, ePrime files containing task trial information, resting state fMRI scans, and, in the case of HCP, fluid intelligence (fIQ) measures. SeeRapuano et al. (2020),section 2.4.1, for more about the inclusion criteria for ABCD. The HCP dataset used in this paper does have data processed with different reconstruction algorithms. Prior work from colleagues Greene et al. suggests that the impact on CPM analyses is negligible (Supplementary Table 3inGreene et al., 2018).

For the HCP dataset, fIQ was measured using a 24-item version of the Penn Progressive Matrices assessment, scores ranged from 4 to 24, with a mean ± standard deviation (SD) of 16.78 ± 4.7 (Bilker et al., 2012). The score corresponds to the number of correct responses.

HCP MRI data were acquired on a 3T Siemens Skyra. The fMRI scans were collected using a slice-accelerated, multiband, gradient-echo, echo planar imaging (EPI) sequence (TR = 720 ms, TE = 33.1 ms, flip angle = 52°, resolution = 2.0 mm^3^, multiband factor = 8). The T_1_-weighted structural scans were collected using a Magnetization Prepared Rapid Gradient Echo (MPRAGE) sequence (TR = 2,400 ms, TE = 2.14 ms, TI = 1,000 ms, resolution = 0.7 mm^3^) (Van Essen et al., 2012).

ABCD MRI data used in this study were acquired using Siemens Prisma or Philips scanners with a 32-channel head coil. Detailed acquisition parameters have been previously described in the literature (Casey et al., 2018). Scan sessions included a high-resolution T1-weighted scan, resting-state fMRI, and task-based fMRI. Functional images for both scanners were collected using an accelerated multiband echo-planar imaging sequence (TR = 800 ms, TE = 30 ms, flip angle = 52°, resolution = 2.4 mm^3^, multiband slice acceleration factor = 6). The Siemens T_1_-weighted structural scans were collected using Magnetization Prepared Rapid Gradient Echo (MPRAGE) sequence (TR = 2,500 ms, TE = 2.88 ms, TI = 1,060 ms, resolution = 1 mm^3^). The Philips T_1_-weighted structural scans were collected using Magnetization Prepared Rapid Gradient Echo (MPRAGE) sequence (TR = 6.3 ms, TE = 2.9 ms, TI = 1,060 ms, resolution = 1 mm^3^).

### 2.2 Preprocessing

For the HCP, the HCP minimal preprocessing pipeline was used on these data (Glasser et al., 2013), which includes artifact removal, motion correction, and registration to MNI space. All subsequent preprocessing was performed in BioImage Suite (Joshi et al., 2011) and included standard preprocessing procedures (Finn et al., 2015), i) with removal of motion-related components of the signal, ii) regression of mean time courses in white matter, cerebrospinal fluid, and gray matter, iii) removal of the linear trend, and iv) low-pass filtering (cutoff = 0.12 Hz).

ABCD data were preprocessed using BioImage Suite (Joshi et al., 2011) with an approach described in detail elsewhere (Greene et al., 2018;Horien et al., 2019;Rapuano et al., 2020). T1-weighted anatomical images were skull stripped using optiBET (Lutkenhoff et al., 2014), and non-linearly registered to MNI stereotaxic space using B-spline free form deformation. Functional images were realigned to correct for motion and registered to MNI space. Further preprocessing was performed as above for the HCP data, including i) removal of motion-related components of the signal; ii) regression of mean time courses in white matter, cerebrospinal fluid, and gray matter; iii) removal of the linear trend; and iv) low-pass filtering (cutoff = 0.12 Hz).

### 2.3 Dynamic connectivity matrix calculation

For both datasets, ROI time series were generated from resting-state and task-based fMRI data using the Shen 268 atlas (Shen et al., 2013) (which defines 268 parcels between cortical and subcortical nodes). HCP data were restricted to one run, in this case the left-right (LR) phase encoded run. The resting-state scan was performed twice in the HCP, and only the first scan was used.

#### 2.3.1 Connectivity measure

Dynamic connectivity matrices were generated from the ROI time series using phase coherence-based connectivity. This method has been demonstrated previously ( Cabral et al., 2017 ; Glerean et al., 2012 ), and has been found to yield similar results to sliding window correlation ( Pedersen et al., 2018 ), though with the advantage of being able to estimate connectivity at single timepoint resolution. In brief, phase coherence dFC was calculated by:

1) Applying the Hilbert Transform to each of the parcellated fMRI time series. This operation splits the time series into two components: magnitude and phase. The phase component was kept.
2) Calculating the pairwise differences of phase components for different ROIs, yielding one vector of phase differences across time for each pair of ROIs.
3) Applying the cosine function to these phase difference vectors, yielding one connectivity matrix of n regions x n regions for each timepoint (268 x 268 for the Shen atlas ROIs).

The matrix values were bounded between 1 and -1, where 1 indicates maximally aligned phases, 0 indicates unaligned phases, and -1 anti-aligned phases. This method is visualized inFigure 1. In all cases, the connectivity matrices generated were undirected, and thus symmetric around the diagonal. As such, 35,778 edges from each matrix were used as features for modeling.

**Fig. 1.**
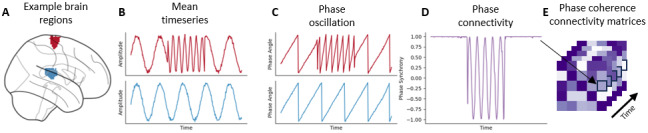
Visualization of the phase connectivity metric. (A) Two example regions of interest from the Shen 268 atlas. (B) Two sinusoids representing example time series, with the frequency of oscillation constant in the bottom time series, and the frequency changing in the top time series. (C) Phase of the example time series, with both time series initially in phase, then rapidly coming in and out of phase, and then in phase again. (D) Phase connectivity of these two time series, with 1 indicating maximally aligned phases, 0 indicating unaligned phases, and -1 anti-aligned phases. (E) Example phase connectivity time series representing one element of a phase connectivity matrix over time.

Static FC is calculated as the Pearson’s correlation (and subsequent z-scores after a Fisher transformation) of the whole time series of each ROI with every other ROI, resulting in a single 268 x 268 matrix for each scan.

#### 2.3.2 Window lengths

To construct windowed connectivity estimates, the mean of dFC estimates at the instantaneous level was taken across the length of the window. The window lengths were decided depending on the target variable. For fIQ prediction, the window length could be arbitrarily set as the fIQ score was independent of the in-scanner task. As such, window lengths of 1, 5, 10, 30, 60, 120, 240, 300, 350, and the full scan were used. For N-Back response time prediction, it was important to take data from when a response was given. In this case, window lengths of 1, 9, 15, 21, and 29 were used, where the middle window timepoint was aligned with the participants’ response. Odd numbers of frames were chosen for prediction of response time so that even numbers of frames from before and after the event could be included. The upper bound window length of 21 timepoints was chosen as it was the largest window possible, whereby the start/end of the scan was not reached. For each window length, all possible data were used irrespective of block type, meaning in some cases there may have been overlap between rest periods and the task block. Windows were allowed to overlap.

#### 2.3.3 Null comparisons

As counterpoints to the standard approach of creating windowed estimates of dFC from scan volumes in the order they were acquired, we also generated dFC from within scan shuffled data. That is, the volumes of each scan of every participant were individually shuffled, rearranging the order of the fMRI volumes. This resulted in data which was misaligned both across participants, with respect to time and task structure. We also generated dFC matrices from a given participant’s resting-state data, without shuffling. These methods are visualized inFigure 2. Each of these different data types was used in the modeling process.

**Fig. 2.**
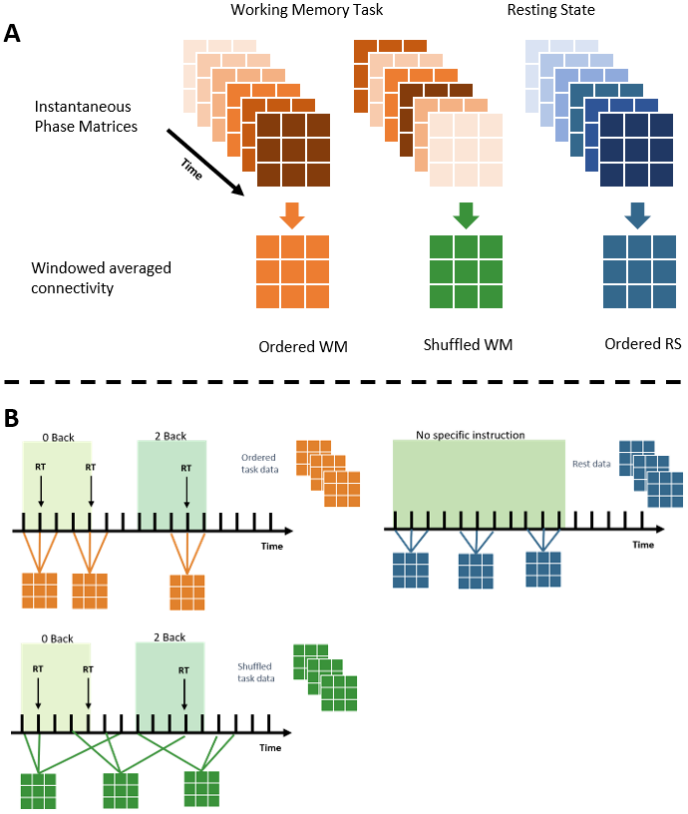
Depiction of how sliding windows were formed from instantaneous phase connectivity estimates. (A) How different instantaneous phase connectivity matrices are combined across time; temporally ordered task fMRI on the left, temporally shuffled task fMRI in the center, and temporally ordered resting state fMRI on the right. (B) Time points forming these connectivity matrices will vary with respect to the task being performed, and the method of combination to form sliding window estimates.

### 2.4 Modeling protocol

#### 2.4.1 Framework

For our modeling framework we chose to use CPM. CPM was performed as in Shen et al. (2017) , with the dFC matrices as the explanatory variable, and either a trait (fIQ) or behavior (N-Back response time, RT) as the target variable, with one exception; partial correlation was used at the feature selection step ( Hsu et al., 2018 ). In full, the CPM process was as follows:

1. Across the training set, correlate each element in the FC matrix, often referred to as an edge (predictive variables) with fIQ (target variable). In this work, partial correlation was used. First, mean frame-to-frame displacement in the fMRI scan is regressed out of both the edge values and fIQ. Then, Pearson’s correlation is computed from the residuals.
2. Select positively correlated edges with, where p < 0.1.
3. For each participant or scan, sum the connectivity scores for all selected edges.
4. Fit a linear model, without regularization, between the sum of connectivity scores and fIQ summary score.
5. Apply the model to unseen participants in the testing set and estimate performance.

This process is visualized inFigure 3, afigure reproduced from a paper byShen et al. (2017). In this study, we performed split half cross-validation (CV) as an initial test of model performance for both fIQ and RT. For RT we evaluated the models further. For this evaluation we aggregated features and model parameters across all the CV folds and created a mean model and feature vector, as described previously inO’Connor et al. (2021). As will be described in more detail below, this mean model and feature vector were evaluated in an in-sample test set, and out of sample.

**Fig. 3.**
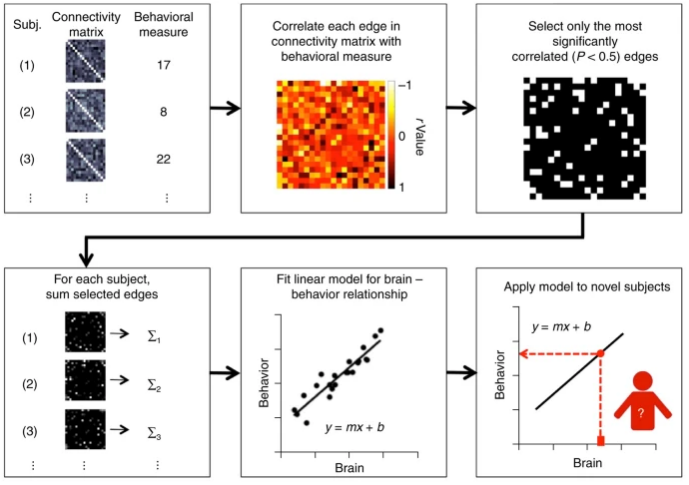
Graphical depiction of connectome-based predictive modeling framework. Reproduced fromShen et al. (2017).

#### 2.4.2 Dataset handling

Following participant exclusion, as described inSection 2.1, the HCP dataset contained 827 subjects, and the ABCD dataset contained 1,668 subjects. These datasets were split into subsets based on our analytic approach. The HCP data were split into two subsamples: a sample of 500, to be used for cross validation-based assessment of the modeling approach, and a separate test set of 327 for evaluating resample aggregated models generated from the first sample. The separate evaluation test set was used exclusively to test within-sample performance, and none of the participants were included in model training.

There were 4 different task block orders for the 1,668 subjects in the ABCD dataset. To facilitate cross-subject comparison, four separate subsamples were created with homogenous task block ordering. This resulted in four subsamples with 426, 393, 402, and 447 subjects respectively, labeled ABCD samples 1, 2, 3, and 4, respectively. The ABCD data were used both for CV-based estimation of RT prediction, and for testing HCP models.

#### 2.4.3 Model training

In the HCP 500 sample, split half CV was performed using dFC to predict both fIQ and RT. Following CV, resample aggregated models were generated for timepoints which showed the highest levels of predictive performance. These aggregated models were then evaluated in in-sample and out-of-sample test sets. For each of the ABCD groups, split half CV was performed using dFC to predict RT. In each case, 50 iterations of split half CV was performed. In the case of the resample aggregated models, this yielded 100 resamples across which to aggregate feature occurrence and model parameters.

#### 2.4.4 Model testing

The HCP test sample was used to assess the within-sample performance of all resample aggregated models generated, and the ABCD data were used to assess out-of-sample performance of these same models. In within-sample testing, each model was tested on 50 random subsamples of ~180 participants from the HCP test sample. In out-of-sample testing, the models were evaluated on 50 random subsamples of 300 participants from each of ABCD samples. We measured the success of a model in terms of R squared, which assesses how much variance a set of predictors or features can account for in a target variable. This choice of metric relates to the main hypothesis of the paper, in that predictive power was used as the measure of feature salience, that is, that brain state specific connectivity matrices may be more salient for certain traits or behaviors than full scan connectivity matrices.

In generating the resample aggregated models, the same approach as detailed inO’Connor et al. (2021)was used. In short, a feature vector was created in which each element corresponded to the frequency with which a given feature passed feature selection, across resampling. This allowed the definition of a minimum frequency threshold, and the exclusion of less frequently occurring features. The resample aggregated models were tested, including features which occurred in 90% or more of resamples. The resample aggregated models’ performance was assessed within- and out-of-sample for each data split, as described above. This framework yielded 50 measures of performance for each model, within-sample, and 50 measures of performance for each model, for each sample of ABCD data, out-of-sample. Model performance was assessed using R^2^.

All analyses were performed in python using custom scripts. These scripts have been made publicly available; see the section on data and code availability. Figures were generated in python using*plotly*,*nilearn*,*matplotlib*(Hunter, 2007), and*seaborn*(Waskom et al., 2017).

## 3 Results

### 3.1 Prediction of trait

**Fig. 4.**
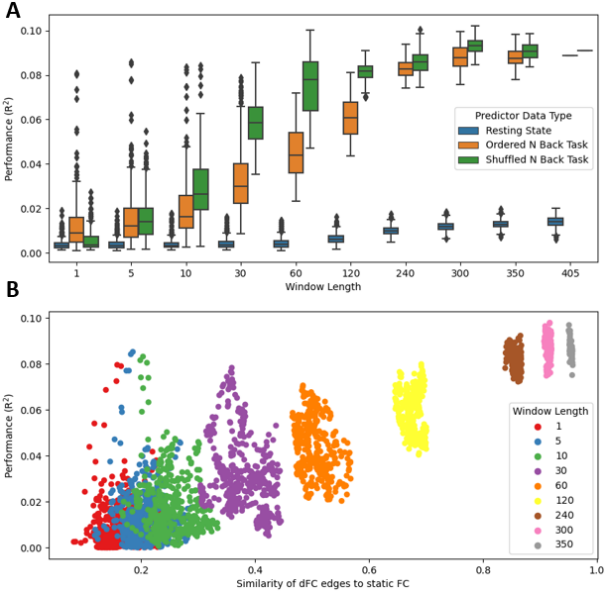
Performance of dFC models across time windows, for prediction of fIQ. (A) Predictive performance (y axis) of resting state dFC (blue), shuffled task dFC (green), and ordered task dFC (orange), for different window lengths (x axis). Data were drawn from across the entirety of the N-Back scans, and the first 405 frames of each subject’s corresponding resting-state scan (matching the number of volumes in the N-Back scan). (B) Performance of ordered task dFC (y axis) versus the similarity of short-time dFC features to features derived at the static level. Window lengths are labeled by color, see legend.

We first attempted to predict a trait, fIQ, from dFC (Fig. 4A). Window lengths ranging from 1 timepoint (instantaneous connectivity) to the full scan length were used in prediction. As a point of comparison, both shuffled task data and resting-state data were used to predict fIQ as well. The first comparison (shuffled task data) was made to ascertain whether the temporal ordering of the data matters. The second comparison (resting data) was made to ascertain whether data without task-driven cognitive processes, but similar noise processes, have predictive power. As shown with the task data, it is possible to explain 2–3% of variance in fIQ, with relatively little data (a window length of 30 timepoints), on par with expected effect sizes of static resting-state FC (Marek et al., 2022). Though the more data included, the better the performance. Interestingly, in the range of 10–120 timepoint window lengths, shuffled task data tended to outperform temporally ordered task data, with its median performance explaining 1–3% more variance in the scores. This is notable given the highest median performance for task-based prediction of fIQ in this study was around 9% variance explained. While it is possible to achieve the same model performance as resting-state data for fIQ from relatively small amounts of temporally ordered task data, better performance is achieved when volumes are drawn randomly from across the whole scan, or when more data are included. This suggests that there are not optimal, temporally specific brain states in isolated parts of the scan which explain fIQ variance better than data from the whole scan. Both task-based prediction methods outperformed resting state for all window lengths.

While the performance of short window dFC never rose to the levels of static FC, we were also curious if the features being selected in short-time dFC models were distinct from those selected by the static model, which may suggest that different parts of the scan reflect different brain states. To assess this, we contrasted the features selected in each of the dFC models with those selected in the static model. This was done by generating mean feature vectors for each timepoint prediction, for each window length in the ordered task data, and correlating them with the mean feature vector from the static FC modelling approach (Fig. 4B). This analysis showed that the features extracted at the dynamic level tended to perform better when they were more similar to the features extracted from the static connectivity matrix. This suggested that short-time scale predictive features provide little benefit in modeling the time-invariant trait.

### 3.2 Prediction of time-varying behavior

#### 3.2.1 Cross-validation: dFC across the scan

**Fig. 5.**
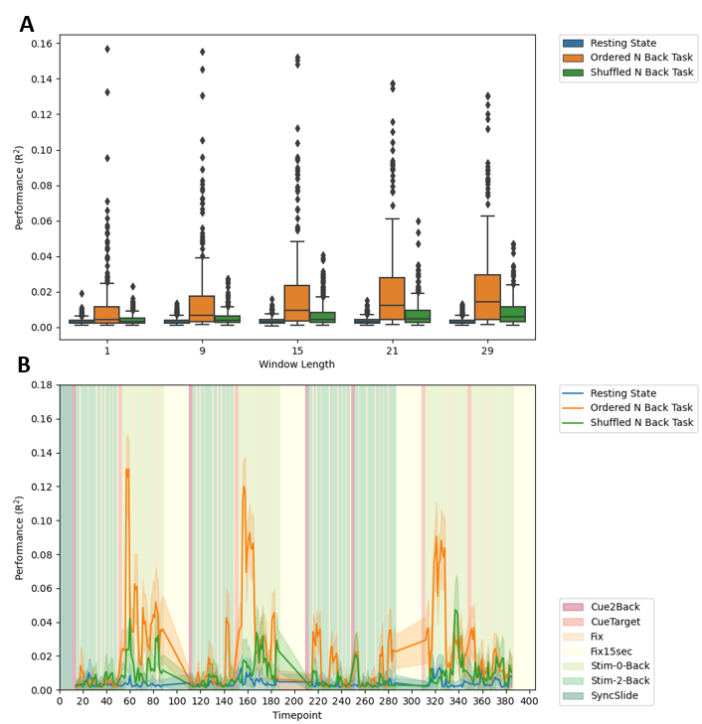
Performance of dFC models throughout the scan, for prediction of response time. (A) Predictive performance (y axis) of resting state dFC (blue), shuffled task dFC (green), and ordered task dFC (orange), for different window lengths (x axis). (B) Performance of each dFC type throughout the N-Back scan, for a window length of 29 timepoints only. The background reflects the N-Back task blocks, as shown in the legend in the lower right legend. The mean performance for the models based on each of resting state dFC (blue), shuffled task dFC (green), and ordered task dFC (orange) is represented by the darker colored line, with the lighter shadowing around the line representing the variance due to repeated model assessment on subsamples of the test set.

Following the above analysis, and acknowledging that traits do not vary within scan, we next sought to test the hypothesis that a behavioral measure, related to a given trait of interest, and which varies across a scan, may be better suited to capture temporally specific brain states. As such, we attempted to build models relating dFC and response time to the N-Back task, a behavior which can vary substantially within an individual’s scan, and across individuals, but which is still related to working memory (Cochrane et al., 2019;Meiran & Shahar, 2018). Using the same approach as for trait estimation, we found that median model performance, across all timepoints, was modest (Fig. 5A). Of note, the performance of shuffled data never exceeded the temporally ordered data, even at a window length of 29, which was comparable to the window length of 30 in the fIQ analysis, where shuffled FC exceeded ordered FC. Median performance of the ordered task model outperformed rest at all window lengths.

#### 3.2.2 Cross-validation: dFC at specific timepoints

While the median performance levels across scan were relatively low, we found there was a substantial difference in prediction performance across the scan for response time. This is reflected in the number of outliers in the box plots inFigure 5A, and is shown more explicitly, for a window length of 29 timepoints inFigure 5B. At certain points in the scan, dFC could explain ~8–13% of variance in the response time of individuals. The start of the 0 back blocks appears to have been optimal, and at these points the model performance exceeded that of predicting fIQ from an equivalent amount of data, whose performance was ~3% of variance for order task data, and ~6% of variance for shuffled task data. Indeed, the dFC-based prediction of response time exceeded even static FC-based prediction of fIQ. In comparing different data types used in the dFC prediction, ordered task data greatly exceeded the shuffled task data, whose peak performance was around 4% variance explained. This suggests that the dFC at these specific points was maximally related to response time, compared to data from other parts of the scan. This also suggests that within scan behavior, and dFC may be highly suitable for recovering time-specific spatiotemporal states. InSupplementary Figures S1–S4, we replicate the plot shown inFigure 5Busing each of the window lengths described inFigure 5A(1, 9, 15, and 21 timepoint windows). The pattern of model performance persisted across timescales, including, most notably, at the instantaneous level. As a further validation of the task-ordered data prediction performance, we also used static FC to predict the response time at each trial. While we recovered a slightly similar temporal profile of model performance, the performance values were much lower; seeSupplementary Figure S5.

#### 3.2.3 In-sample replication of dFC at specific timepoints

To assess the reproducibility of our findings thus far, specifically the high levels of dFC-based model performance for response time, we tested how the best performing models, occurring at timepoints 59, 158, and 325, performed in novel subjects in the HCP test sample. To do so, we aggregated features and model parameters across CV folds and iterations, and generated resampled models as in previous work (O’Connor et al., 2021). We applied a 90% threshold to the feature vector, that is, selecting only the features occurring in 90% or more folds across iterations. We ended up with three feature vectors, and three corresponding linear models. Again, using the CPM framework to predict response time, we applied each of the models, separately, to within-sample data which had been completely omitted from the training process. We ended up with the three temporal profiles of model performance shown inFigure 6. Each of the models performed best around the timepoints they were generated, and in each case better than the other two models. This further suggests a temporal specificity to the features extracted. Altogether, these analyses suggest that when using dFC in predictive models, temporal structure matters in modeling brain-behavior relationships, and time-varying behavior-based analyses are more suitable for finding temporally specific brain states than trait-based analyses.

**Fig. 6.**
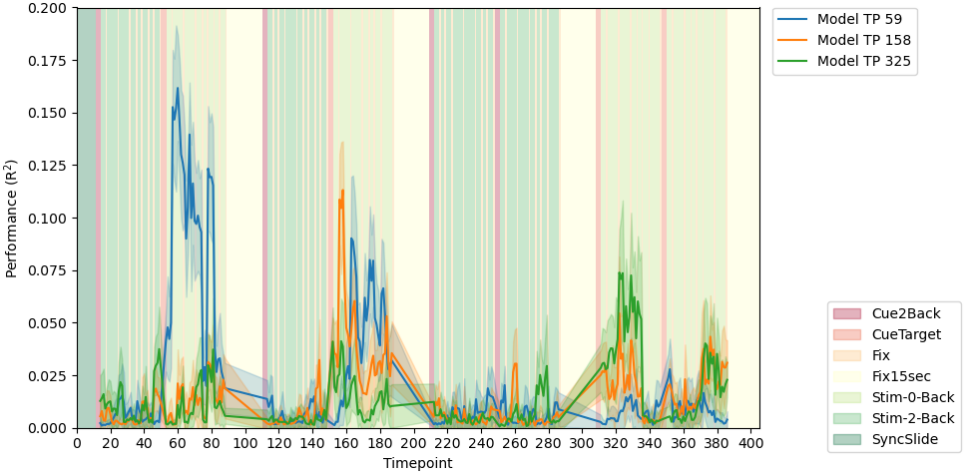
Within-sample performance of resample aggregated models dFC and response time, generated from highest performing timepoints of the N-Back HCP task. Performance is shown for features which were selected in 90% of folds across 50 iterations of split half cross-validation. The background coloring reflects the task blocks, as shown in the legend in the lower right. The mean performance for each of the models based on timepoint 59 (blue), timepoint 158 (green), and timepoint 325 (orange) is represented by the darker colored line, with the lighter shadowing around the line representing the variance due to repeated testing on subsamples of the test set.

#### 3.2.4 Model features of timepoint-specific dFC models

The feature vectors used above are shown inFigure 7. Across the three models, visual, motor, limbic, and frontoparietal networks contain the greatest number of edges, consistent with task demands and previous work (Yaple et al., 2019). Model 59 has the most distributed set of edges, with many brain regions contributing to the model performance. Models 158 and 325 show increasing dependency on fewer edges, with the edges in model 325 coming mainly from four networks pairs (visual association-frontoparietal; visual association-limbic; visual network II-frontoparietal; visual network II-limbic). At a 90% threshold, there are no overlapping edges between the models. A similar plot toFigure 7is shown inSupplementary Figure S6, with a lower threshold (30%) to illustrate the less frequently occurring edges, where more overlap occurs. Also shown are histograms of edge occurrence frequencies inSupplementary Figure S7.

**Fig. 7.**
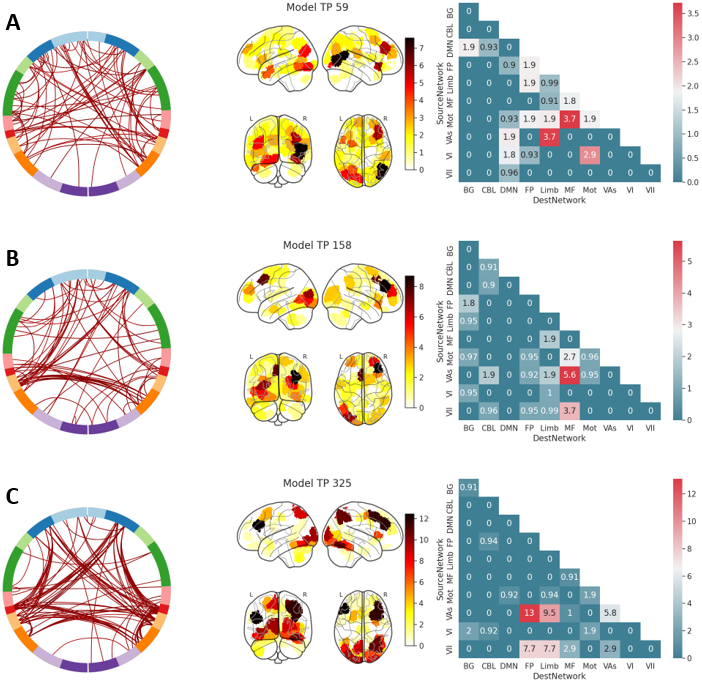
The feature sets from the highest performing models, as depicted inFigure 6. Shown are feature sets for the models from timepoints 59 (row A), 158 (row B), and 325 (row C). Feature sets were generated at a 90% feature frequency threshold. The first column shows a circle plot, which displays an edge-by-edge visualization of the features. The second column shows a degree plot, which is what regions were present in the model weighted by their frequency of occurrence in an edge pair. The third column shows a network-level matrix of what networks were represented in the feature vector, also weighted by their frequency of occurrence, and normalized for number of edges within network. The circle plot labels match the following brain networks: MF: Medio Frontal, FP: Front Parietal, DMN: Default Mode Network, Mot: Motor, VI: Visual I, VII: Visual II, VAs: Visual Association, Limb: Limbic, BG: Basal Ganglia, CBL: Cerebellum.

#### 3.2.5 Generalizability of timepoint-specific dFC models, out of sample

**Fig. 8.**
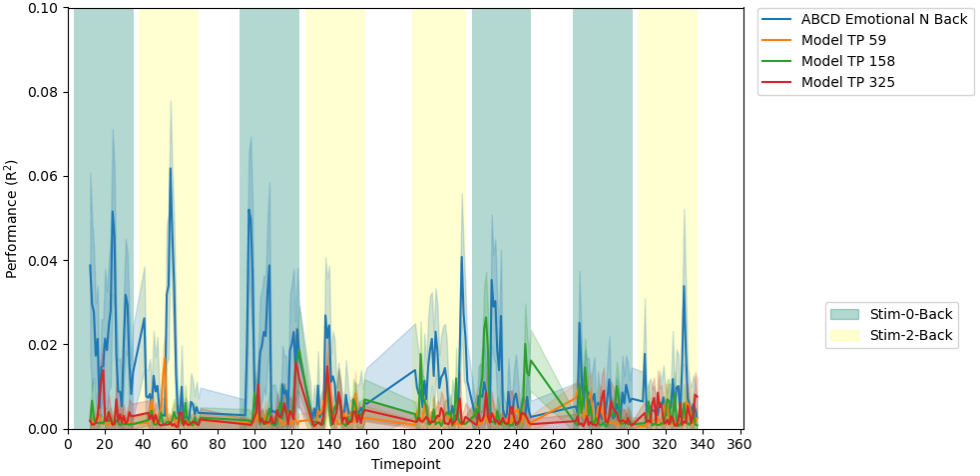
Performance of the HCP models, out of sample (orange, green and red), juxtaposed with prediction performance of models built from ABCD data (blue). These performances are from “group 4” of the ABCD. Similar plots for the other three groups are shown inSupplementary Figures S7–S9. The background reflects the task blocks, as shown in the legend in the lower right. The mean performance for the model based on each of ABCD Emotional N-Back (blue), HCP N-Back timepoint 59 (orange), HCP N-Back timepoint 159 (green), and HCP N-Back timepoint 325 time is represented by the darker colored line, with the lighter shadowing around the line representing the variance due to testing on repeated subsamples of the test set.

As a final test of model performance, the three models discussed above (from timepoints 59, 158, and 325) were applied to ABCD task data, specifically the emotional N-back task (Fig. 8). The ABCD scan protocols had slightly lower temporal resolution, so a window length of 25 timepoints was used to ensure similar lengths of scan time were covered. The models, which were built with HCP data only, show negligible performance. Also shown, as a point of reference, is the cross-validated performance of models built from the ABCD data, using a similar method to the above. The performance of the ABCD-based models does not reach those of the HCP, though they can explain 2–6% of the variance in response time at different points in the scan, again on par with expected effect sizes of static resting state FC (Marek et al., 2022). The pattern observed in the HCP, of high performance at the start of zero back blocks, is not replicated. As the ABCD task block ordering varied across participants, we created four groups of participants with similar task block ordering.Figure 8shows data from group 4, groups 1–3 are shown inSupplementary Figures S7–S9. Groups 1 and 3 show similar patterns to group 4, in that the models built in the HCP exhibit low performance, and with high variability across time, while the ABCD-based cross-validated models exhibit better performance in the range of 2–6% variance explained. Group 2 shows middling to poor performance for all models.

## 4 Discussion

### 4.1 Summary

In this study, we employed a regression model-based approach to identify trait and behavior-related brain states using phase synchrony based dFC. We contrasted rest- and task-based fMRI, as well as trait and behavior, and showed that task-based prediction of time-varying behavior may be more optimal for identifying temporally specific brain states. We assessed the models derived using cross-validation, within-sample testing, and out-of-sample testing. We found that response time to an N-Back task could be optimally predicted at the start of zero back blocks, in three specific time periods, representing three different brain states with different model features. We also found that resampled response time models generated during these brain states replicated well within samples around the sample timepoints where they were built. This dFC-based approach for model building showed success in the ABCD sample. This framework has the potential to be useful in brain state-based investigation of time-varying behaviors in the future.

### 4.2 dFC versus static FC, brain states

We examined a wide range of window lengths for fIQ prediction and a more limited range of window lengths for response time prediction. Our main dFC results for response time prediction focused on a window length of 29 timepoints, the equivalent of 20.88 s of data, which is at the lower end of traditional dFC analyses. However, we also saw high levels of predictive performance, for specific timepoints, at multiple temporal scales. Our initial cross-validation analysis, using behavior-based prediction, was able to capture temporally specific and predictive features at each time scale. Model performance persisted for all window lengths, including at the instantaneous level; seeSupplementary Figures S1–S4. It was also possible to predict fIQ from short-time dFC, though the performance levels never exceeded those of static FC, and the predictive features tended to be more similar across the scan.

dFC allows for more temporally precise measurement of FC, with the possibility of investigating temporally specific aspects of functional brain connectivity during scanning. Traditionally, dFC-based studies of brain states have used longer time windows to estimate connectivity, in the range of 40–100 s (Leonardi & Van De Ville, 2015;Zalesky & Breakspear, 2015), often with longer repetition times than the protocols used in this study. There are studies which have shorter window sizes (Cabral et al., 2017;Gonzalez-Castillo et al., 2015), or no window at all (Yaesoubi et al., 2018). However, these studies still aggregate data from the whole scan into the derivation of brain states. Notably, our regression-based approach, based on phase synchrony dFC, can go beyond more typical sliding window methods, and allow for estimation of brain states using very little data per participant, down to the level of individual timepoints. When combined with short TR acquisitions, this allows for high temporal specificity. It is important to note that window length will inherently have trade-offs with respect to variability of FC measurements and the overall temporal stability (Lurie et al., 2020) (see ‘Limitations and Future Studies’ for more).

There is precedent for predicting behavioral and perceptual outcomes from short-time FC, though without the explicit focus on brain states. An early study in the field byG. J. Thompson et al. (2013)modeled response time to a psychomotor vigilance task using regression, based on short-time connectivity estimates from two brain networks. Classification approaches have also been used to predict auditory (Sadaghiani et al., 2015) and tactile stimuli (Weisz et al., 2014), as well as preference for faces (Bhushan et al., 2012).Kucyi et al. (2017)showed that “response times” to a self-paced task, characterized by repetitive button pressing, could be used to determine periods of fatigue or distraction. Working memory specific reconfiguration of dFC has also been well documented (Braun et al., 2015;Shine et al., 2016;Vatansever et al., 2015). This study is well placed among these findings, given that an individual’s response time to an N-Back task likely incorporates many phenomena, including perception, attention, and fatigue. Our study was unique in that it combined a regression approach, an order of magnitude more of subjects than some of the studies listed above, a parcellation scheme with whole-brain coverage, and only ~20 s of data or less per participant for any given model. We assert that the method detailed here is relevant both to the study of behaviorally related brain states and for short-time predictive modeling.

### 4.3 Resting state versus task, nulls

We sought to find temporally specific spatial patterns of brain connectivity which were related to a trait or behavior of interest. To assess the utility of task-based dFC, we compared the predictive power of (a) temporally ordered task-based fMRI, (b) temporally shuffled task-based fMRI, and (c) resting state fMRI. Previously, it has been shown that static resting state FC is a poorer predictor of fIQ than task-based connectivity (Greene et al., 2018), though it was unclear if this would hold for dynamic connectivity, or for response time prediction. Here, we confirm that resting-state connectivity is a poorer predictor of fIQ at the dynamic level, and its predictive value remains negligible until 4–5 min of data are included. A similar trend is seen for response time. We suggest that resting state fMRI, within the context of dynamic assessment of behaviorally related brain states, can provide a useful foil for task-based dFC, serving as a null space for comparisons. Indeed, it has the same nuisance factors as task-based data, while the cognitive content is more spontaneous and uncoordinated (Kucyi, 2018;Smallwood et al., 2021). Another comparison conducted was whether temporally ordered task data led to better predictive models than randomly ordered, or shuffled, task data. We found that shuffled task data performed better for trait prediction, while temporally ordered data worked better for behavior prediction. This suggests that for trait-based predictions, drawing data from across the scan is advantageous, whereas, for behavior, more precise estimates of connectivity are better. Cross-subject synchrony, or lack thereof, is an important aspect in FC-based analyses, and synchrony and desynchrony may reflect different, but equally cognitively relevant processes (Nastase et al., 2019).

As well as wanting to assess the biological relevance of time-ordered task data, we also sought to bolster our confidence in the results of our analytic methods. As discussed in the introduction, a common method in dFC research is the construction of null spaces, simulating random data from real covariance matrices and power spectra (Laumann et al., 2017;Miller et al., 2018). The goal of these efforts is to interrogate the validity of the results drawn from real fMRI data, in particular resting-state data. However, it is difficult to fully replicate real physiological noise, and motion, present in these data. We used resting state dFC as our “null”. Task-based dFC analyses, which rely on cross-subject synchrony, can be juxtaposed with a resting dFC to provide a rigorous statistical comparison. This approach may prove advantageous in harnessing dFC for use in a clinical setting where a particular trait or behavior of interest that is to be interrogated with a task paradigm can be interrogated with a task paradigm while using the same subject’s resting-state data as the null condition.

### 4.4 Modeling and generalizability

We derived three regression models of time-varying behavior, which replicated within sample, on unseen subjects. The resampling technique used to generate mean feature vectors allowed us to assess how much variance there is in feature selection within sample. Boiling down feature sets to the most salient features, that is the ones which occur most frequently, and assessing on unseen data offers a path forward to interrogating and replicating the success of brain-behavior models (O’Connor et al., 2021). Generalizability is a key roadblock in the development of brain-behavior models, with overfitting the norm rather than the exception. The evaluation of models in out of sample data can be difficult. Failure to replicate within sample would indicate complete overfitting, but one can interpret a lack of generalization to other datasets in different ways. This topic has been covered previously (Yarkoni, 2019), including in our own work (O’Connor et al., 2021). It is possible that a model can capture salient features reflecting underlying cognition, while failing to generalize. The ABCD data comes from a multisite on-going initiative, presenting possible confounds that may arise due to multiple scanner and site differences (Badhwar et al., 2020). This is in addition to sample based phenotypic differences, age being a prime factor which impacts working memory performance (Cowan et al., 2011), and task paradigm differences. The N-Back was used in both HCP and ABCD, but the ABCD used different stimuli, designed to evoke emotional responses, in addition to testing the working memory of participants (Casey et al., 2018). Each of these factors could impede generalization across these datasets. It may be that case that the cognition underlying response time to a working memory N-Back task in adults, as represented by dFC, is fundamentally different to response time to an emotional N-Back task in 9–11-year-olds. That is to say, perhaps the dataset we chose to attempt generalization in was not the ideal test case. Nonetheless, it is imperative to assess the degree to which model generalization is impeded and attempt to overcome these differences. In that vein, we hypothesized that by reducing the amount of data taken from a given dataset, we may be able to capture only the most relevant parts of a study, potentially aiding generalizability. While between study differences may have contributed to a lack of transfer of models between studies, the ABCD data did validate our approach to model building, as we could generate within sample models in both data sets, which explained variance in N-Back response time.

### 4.5 Trait versus behavior

So far, we have focused on the imaging aspect of the brain-behavior modeling. The terms trait and behavior are often used interchangeably; in this case, we consider traits to be either unchanging, or changing over much longer periods of time, when compared to time-varying behavior which can change moment-to-moment (Rosenberg et al., 2020). Often, traits are used as axes of contrast across participants in dFC analyses, for example a diagnosis, or a score on a cognitive assessment. Aside from the fact that these are not permanent attributes, and may vary across different periods of time, they do not vary over the length of a scan. We would argue that this makes them poor candidates for brain state-based dFC analyses. A better approach may be to incorporate a manipulatable behavior, related to the trait under consideration. As mentioned above, reproducibility and generalization should be of major concern in any study. A recent paper byElliott et al. (2021)proposes several methods for conducting better studies, including i) aggregating more repeated in-person measurements, ii) modeling points of the scan with stable variability, iii) better accounting of physiological noise, and iv) designing more reliable tasks. The framework described above can be used to address points ii) and iv) by identifying stable brain states which have sustained relationships with behaviors of interested, as assessed via continued high performance of the model. This can then be used to probe the usefulness of a task fMRI paradigm at the maximum allowable temporal resolution. This opens the possibility of a feedback loop between task design and model performance, whereby tasks can be designed to maximize model performance or effect size. Maximizing the effect size for the studied relationship is particularly relevant in the context of how effect size and sample size affect the power, and therefore reliability, of a study (Geuter et al., 2018;Grady et al., 2021;Marek et al., 2022;Noble et al., 2021).

### 4.6 Limitations and future studies

In this study, we did not utilize a method for site harmonization. We sought to find features which might naively generalize to other sites, in this case from the HCP data collection site to the ABCD group of collection sites. To aid cross-site comparison, the ABCD sites underwent extensive harmonization efforts (Casey et al., 2018). Recent work by Marek et al. showed that sampling variability was equivalent between the single-site HCP, and multi-site ABCD; see extended data figures 6a and 6b inMarek et al. (2022). However, this study found it was still a challenge to generalize across HCP and ABCD, and future work may benefit from site harmonization efforts (Badhwar et al., 2020;Yu et al., 2018). We also made the choice to focus on brain regions which positively correlated with the target variable, and excluded those which negatively correlated, to simplify assessment of the analytic methods. A future paper may choose to incorporate both positively correlated and negatively correlated features in the same study.

Another methodological choice was to use a previously documented preprocessing pipeline (Papademetris et al., 2006). We have found this method to be effective in uncovering brain-behavior relationships in prior static connectivity-based studies (Finn et al., 2015;Gao et al., 2019;Greene et al., 2018;Hsu et al., 2018;S. Ju et al., 2023;Y. Ju et al., 2020;O’Connor et al., 2021). In addition, the pipeline has been leveraged to build generalizable brain-behavior models (Horien et al., 2022;Rosenberg et al., 2016;Yoo et al., 2018) and has also been used in multi-site studies to determine robust markers (Lake et al., 2019;Rapuano et al., 2020;Rohr et al., 2020). Assessing the effects of different preprocessing strategies on short-time connectivity methods was beyond the scope of the current study, but is worth investigating given the demonstrated impact they can have on static connectivity-based studies (Ciric et al., 2017;Lindquist et al., 2019;Parkes et al., 2018). Similarly, to preprocessing methods, the choice of atlas can have downstream effects on the study outcome although they are small in CPM because of the large number of edges selected. The choice of window length, and indeed where to sample the window with respect to temporally varying behavior, is also an important consideration. We made the choice to center the sliding windows around behavioral responses, drawing equally from before and afterward. Future studies could assess how different sliding window choices affect model performance. We hypothesize it would vary significantly based on the behavior being studied and to a lesser degree on the temporal and spatial resolution of the data.

## 5 Conclusion

We have developed a framework for identifying behaviorally relevant short-time brain states, as represented by dynamic functional connectivity maps. We find that drawing data from across the scan is better for trait-based prediction, but more isolated and temporally specific parts of the scan are better for developing models that predict in-task performance. The resample aggregated dynamic functional connectivity models of behavior replicated within sample using unseen HCP data, and this same modeling framework showed success in estimating the variance behavior in the ABCD dataset. Future work could assess if the framework described here could be used to model other time-varying behaviors under different task conditions.

## Supplementary Material

Supplementary Material

## Data Availability

The HCP data that support the findings of this study are publicly available on the ConnectomeDB database (https://www.humanconnectome.org/). The ABCD data used in this report came from NIMH Data Archive Digital Object Identifier 10.15154/1504041. DOIs can be found atnda.nih.gov/study.html?id=721. Code for conducting the analyses described here can be found athttps://github.com/YaleMRRC/dFCAnalysis
